# A parallel epidemic of sino-nasal mucormycosis post-COVID pneumonia, and its anesthetic implications: a case report

**DOI:** 10.1186/s42077-023-00327-5

**Published:** 2023-05-16

**Authors:** Prateek Arora, Abhishek Bharadwaj, Omer Mohammed Mujahid, Monica Khetarpal

**Affiliations:** Department of Anaesthesiology, Pain & Critical Care, All India Institute of Medical Sciences, GE Road, Raipur, Chhattisgarh 492099 India

**Keywords:** Mucormycosis, SARS-CoV-2, COVID pneumonia

## Abstract

**Background:**

There has been a rise in cases of sino-nasal mucormycosis in patients who contracted the COVID-19 virus and were on steroids. Population at risk includes diabetics and on immunosuppressant therapy and/or immunocompromised state. Perioperative management becomes challenging due to residual pulmonary disease secondary to COVID pneumonia and complication following systemic antifungal therapy. Such patients often have other associated illnesses like hypertension, obesity, and deranged renal functions, either as a part of metabolic syndrome or post-systemic antifungal therapy.

**Case presentation:**

We report a case of a 64-year-old female, a poorly controlled hypertensive, with class 1 obesity, and diabetic on oral hypoglycemic agents, hypothyroid on oral medications, and post-COVID pneumonia with recently diagnosed sino-nasal mucormycosis who was posted for debridement of the sino-nasal fungal mass. The gamut of co-morbid conditions along with post-COVID pneumonia status presents an anesthetic challenge apropos the optimization of the clinical conditions and timing of the surgery considering the emergent nature of the surgery.

**Conclusions:**

The timing of operative intervention for such patients is imperative and the treating team of anesthesiologist and the otorhinolaryngologist should aim to strike a balance between timely intervention to negate the spread of the infection to the orbit and brain causing potential irreparable damage and optimizing the cardio-respiratory and renal functions.

## Background

The surge in COVID pneumonia cases has brought about a wave of fungal infections with it especially in patients with diabetes, on immunosuppressive therapy, use of steroids and immunosuppressive state. Zygomycosis fungal infection can affect the nasal cavity and paranasal sinuses and can spread into orbit and the cranial cavity, possibly leading to catastrophic events. Many such patients need operative interventions for debridement of the fungal lesion and administering anesthesia for such patients can be challenging due to various comorbidities and the effect the disease and the medications cause on various organ systems of the body as well as mental health of the patient. It is often the case that patients cannot be fully optimized before undergoing anesthesia. The case in question highlights one such example of having multisystem involvement needing timely intervention.

## Case presentation

**Fig. 1:**
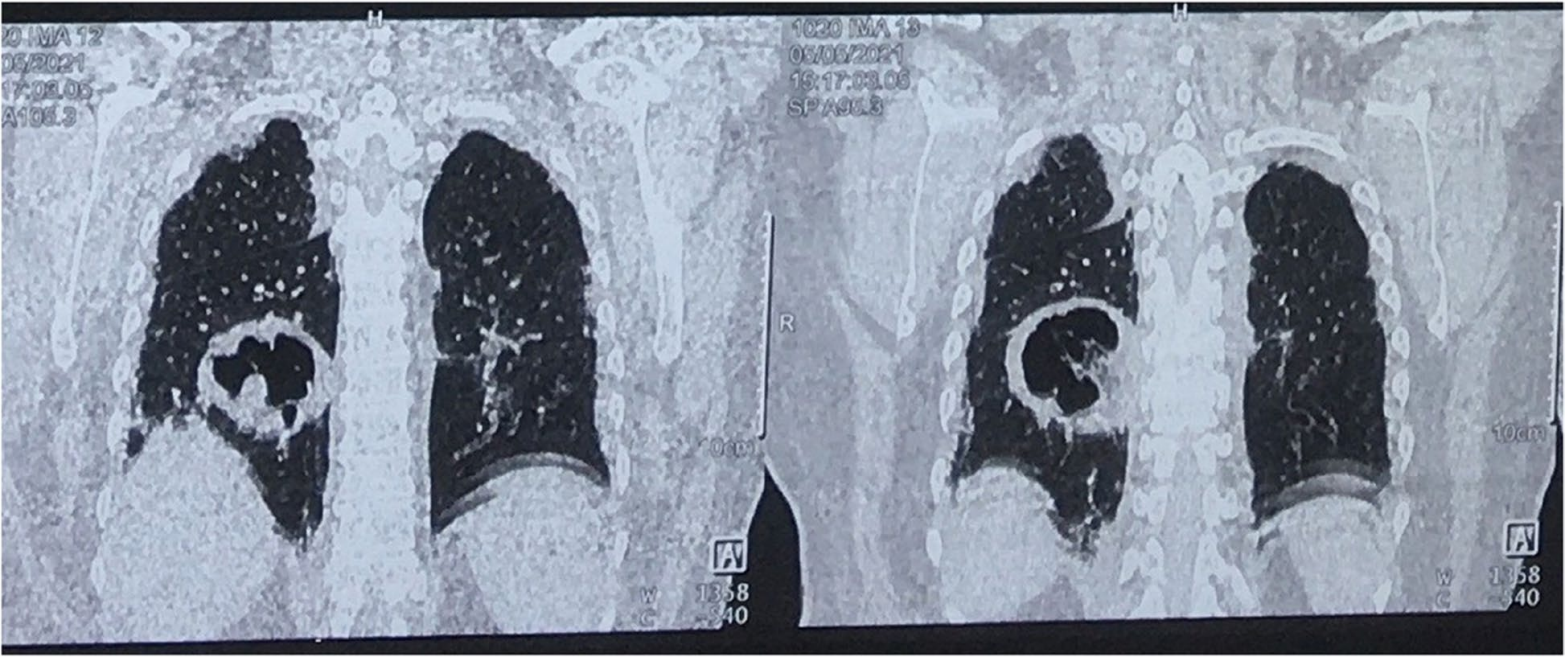
High-resolution CT lung showing multifocal fibrotic changes, honeycombing, and bronchiectasis. Cavitary lesion in the posterior basal segment of the right lower lobe

**Fig. 2:**
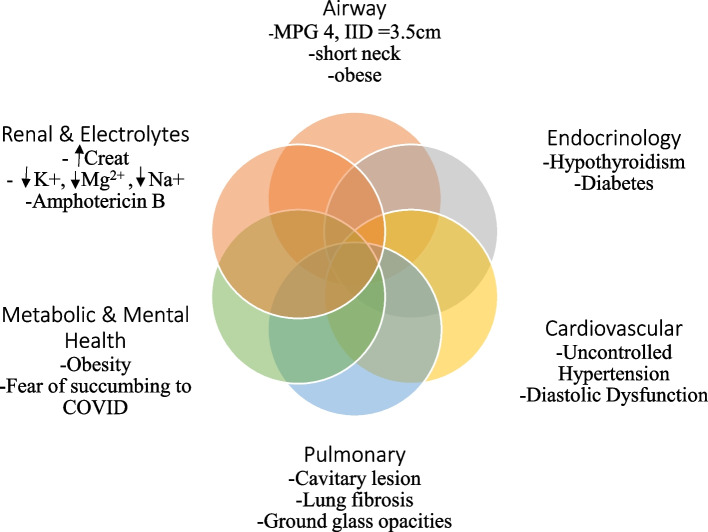
Diagram showing the systemic involvement and concerns in the patient with sino-nasal mucormycosis post-SARS-CoV-2 pneumonia, undergoing surgery under general anesthesia

A 64-year-female presented to the emergency department with complaints of fever and headache since 15 days, two episodes of epistaxis 10 days back, and pain and swelling around the right eye for 5 days, which was insidious in onset and gradually progressive; the pain increased on movement and in lateral gaze. It was associated with redness and yellowish discharge from the eye. She was a known hypertensive for 15 years with poor compliance to medicines, class 1 obesity with a BMI of 32 kg/m^2^. She was a diabetic for the past 10 years, with poor glycemic control on OHAs (HbA1C = 8.2). She also had hypothyroidism and was on tab thyroxine 50 mcg daily (TSH = 5.11 IU). The patient gave history of taking medications prescribed through a local dispensary and over-the-counter drugs for her condition, before coming to the district hospital. On admission there, she tested positive for SARS-CoV-2 and her vitals were pulse rate 110/min, BP 180/90 mmHg, saturation of 93% on room air, respiratory rate of 34/min, and temperature of 101°F. She was given primary treatment with antibiotics, steroids, antihypertensives, antipyretics, and fluid at the district hospital and was referred to a higher center. On arrival at our institute, she was admitted in the COVID facility and was maintaining a saturation of > 96% on non-rebreathing mask with oxygen at 10L/min and was started with supportive measures for COVID pneumonia and measures to optimize her blood pressure and blood glucose levels were undertaken. Investigations revealed a microcytic hypochromic anemia with hemoglobin of 8.1 g/dL and a WBC count of 10.47 μl^−1^. Her renal functions showed a serum urea of 56 mg/dL, creatinine of 2.1 mg/dL, Na + 126 meq/L, potassium 2.8 meq/L, and magnesium 1.24 mg/dL. The inflammatory markers showed ferritin > 1650 ng/mL, IL-6 22.9 pg/mL, CRP 49.25 mg/L, and ESR 96 mm.The ECG depicted normal sinus rhythm, normal axis, low QRS voltage, and T wave inversion in Lead 1 and aVL. 2D-echocardiography showed concentric left ventricular hypertrophy, no RWMA, an ejection fraction of 60%, moderate diastolic dysfunction (E/A 1.3) trivial TR, and RVSP 17.35 mmHg. Chest imaging showed features suggestive of multifocal fibrotic changes, early changes of honeycombing, and bronchiectasis (Fig. 1). Cavitary lesion measured 4.9 × 6.2 × 6.3 cm in the posterior basal segment of the right lower lobe, with segmental bronchus seen to course along the wall of the lesion. The fungal cultures obtained from the pus discharge revealed the growth of mucormycosis. She began receiving systemic antifungals at a dosage of 5 mg/kg/day of liposomal amphotericin B. Patient perceived an increase in eyeball swelling after a brief initial reduction in size following treatment. This was confirmed by the treating otorhinolaryngologist, and a debridement of the fungal mass was planned under general anesthesia. Perioperatively, measures were taken to tailor the anesthesia plan based on the patient’s condition, keeping in mind the concerns (Fig. 2). Patient was nebulized with 4% topical lignocaine, prior to shifting to the operating room. The anesthesiologist followed COVID-appropriate protocols during airway management, which included wearing N95 masks, gloves, gowns, and goggles for eye protection. In the operating room, left radial artery cannulation and central venous access were obtained under local anesthesia, in view of difficult peripheral IV access. ASA standard monitors were attached, and the patient was made to lie in a ramp-up position and was preoxygenated. The patient was induced with a sleep dose of propofol and succinylcholine 2 mg/kg, which was used as muscle relaxant. Trachea was intubated with a 7.5-cuffed endotracheal tube using a video laryngoscope. Laryngoscopy response was attenuated using 10-mg aliquots of intravenous esmolol titrated to response. After confirming the tube placement with capnograph, Inj fentanyl 2mcg/kg, Inj atracurium 0.5 mg/kg, stat were given and the anesthesia was maintained on desflurane at MACage 1–1.2 in oxygen and air mixture (40:60) with low flow of 600 mL/min on PCV-VG (Mindray A7) mode of ventilation, with peak airway pressures at 19–20 mmHg, PEEP of 5cmH2O, and tidal volume and respiratory rate set to maintain an end-tidal CO2 concentration of 32–35 mmHg. Fentanyl infusion was initiated at 1mcg/kg/h and a potassium correction was initiated. Intraoperatively Ringer lactate was used as maintenance fluid as per institutional protocol, which was aided by the trend of PPV, hemodynamic parameters, and surgical losses. Thereafter, blood transfusion was initiated in view of low preoperative hemoglobin and to replace the surgical losses, guided by the hemodynamic parameters. Glycemic control was maintained to keep blood sugars between 130 and 180 mg/dL. A POCUS revealed multiple B-lines all over the lung field and small RV cavity. The corresponding PPV on the multipara monitor was 16–17. The ABG obtained 20 min post-induction showed a PCo2 of 46 mmHg and a PO2 of 220 mmHg on FiO2 of 0.4. The surgical team performed a nasal endoscopy to assess the spread of the fungus and evaluate the osteomeatal complex; thereafter, with a lateral rhinotomy incision, the nasal cavity and sinuses were cleared and washed. The patient withstood the procedure well and steady hemodynamics were maintained. A total blood loss of 550 mL was noted. At the end of the procedure, the patient was shifted to the post-anesthesia care unit and the residual neuromuscular blockade was reversed and the trachea was extubated. After observation and fulfilling the Aldrete discharge criteria, the patient was transferred to the COVID critical care unit in the facility.

## Discussion

Initially, SARS-CoV-2 infection was characterized by a dry cough and high fever, but now it encompassed a wide range of symptoms, including shortness of breath, loss of smell or taste, diarrhea, general discomfort, acute cardiac injury, thrombosis, and secondary infections (Sharma et al. 2021). It is advocated that besides the use of steroids and immunosuppressive drugs, SARS-CoV-2 infection by itself can induce an immunosuppressive state that exposes the patient to the risk of developing opportunistic infections, such as molds (Pasero et al. 2020). Chen et al. (Chen et al. 2019) suggested a complex interplay of factors, including preexisting diseases, such as diabetes mellitus, previous respiratory pathology, use of immunosuppressive therapy, the risk of hospital-acquired infections, and systemic immune alterations of COVID-19 infection itself, may lead to secondary infections, which are increasingly being recognized in view of their impact on morbidity and mortality. These kinds of infections by themselves are associated with the worst outcome, especially when the immune system response does not improve. The Global guideline (Cornely et al. 2021) for the diagnosis and management of mucormycosis emphasizes that surgery, control of hyperglycemia, and early treatment with liposomal amphotericin B are essential for the successful management of mucormycosis. However, coexisting ARDS and multiorgan dysfunction preclude timely diagnostic imaging, testing, and intervention (Pasero et al. 2020). The added burden of COVID-19 patients on healthcare takes a toll on other non-COVID essential services including diagnostic tests and surgeries, which in itself could possibly delay the intervention and hamper the outcome (Garg et al. 2021). It is evident that there are numerous anesthesia-related considerations in this scenario, and their effective management presents a formidable challenge. As illustrated in this case report, the patients most often will be far from optimized. The key to crafting an anesthesia plan for such patients is to incorporate lung protective strategies and to prevent renal insult. Ventilatory modes such as the pressure control ventilation-volume guarantee (PCV-VG) guarantees a set tidal volume at the lowest required inspiratory pressure adjusted breath by breath. Ensuring goal-directed fluid therapy ensures renal perfusion and prevents lung flooding. Implementing an individualized, patient-centered strategy that carefully maintains a fine balance of therapeutic interventions can facilitate safe passage through the perioperative period.

## Conclusions

The SARS-CoV-2 pandemic has redefined the health crisis and has taught us that things are not ideal. With an unprecedented surge in cases of mucormycosis post-COVID pneumonia, a lot more patients with multiple co-morbidities will come for procedures of the airway needing general anesthesia. Most often due to multi-system involvement and nature of disease, the condition of the patient will be far from ideal. A strong clinical judgment and a tailored anesthesia plan are needed for such cases to prevent the patients from further deterioration in the perioperative period and give us, the anesthesiologist, as perioperative physicians an opportunity to optimize and treat the patients underlying condition.

## Data Availability

Not applicable.

## Abbreviations

ECG: Electrocardiogram
BMI: Body mass index
OHA: Oral hypoglycemic agents
TSH: Thyroid-stimulating hormone
ESR: Erythrocyte sedimentation rate
CRP: C-reactive protein
RWMA: Regional wall motion abnormality
RVSP: Right ventricular systolic pressure
POCUS: Point Of Care Ultrasonography
PPV: Pulse pressure variation

## References

[CR1] Chen N, Zhou M, Dong X, Qu J, Gong F, Han Y, et al (2020) Epidemiological and clinical characteristics of 99 cases of 2019 novel coronavirus pneumonia in Wuhan, China: a descriptive study. Lancet 395(10223):507–13. 10.1016/S0140-6736(20)30211-7. [cited 2021 May 11] 10.1016/S0140-6736(20)30211-7 PMC7135076 32007143

[CR2] Cornely OA, Alastruey-Izquierdo A, Arenz D, Chen SCA, Dannaoui E, Hochhegger B, et al (2019) Global guideline for the diagnosis and management of mucormycosis: an initiative of the European Confederation of Medical Mycology in cooperation with the Mycoses Study Group Education and Research Consortium. Lancet Infect Dis 19: e405–21. Available from: https://pubmed.ncbi.nlm.nih.gov/31699664/. [cited 2021 May 11]. 10.1016/S1473-3099(19)30312-3 PMC8559573 31699664

[CR3] Garg D, Muthu V, Sehgal IS, Ramachandran R, Kaur H, Bhalla A, et al (2021) Coronavirus disease (Covid-19) associated mucormycosis (CAM): case report and systematic review of literature. Mycopathologia 186(2). Available from: https://pubmed.ncbi.nlm.nih.gov/33544266/. [cited 2021 May 9]. 10.1007/s11046-021-00528-2 PMC7862973 33544266

[CR4] Pasero D, Sanna S, Liperi C, Piredda D, Branca G Pietro, Casadio L, et al (2020) A challenging complication following SARS-CoV-2 infection: a case of pulmonary mucormycosis. Infection. Available from: https://pubmed.ncbi.nlm.nih.gov/33331988/. [cited 2021 May 9]. 10.1007/s15010-020-01561-x PMC7745708 33331988

[CR5] Sharma S, Grover M, Bhargava S, Samdani S, Kataria T (2021) Post coronavirus disease mucormycosis: A deadly addition to the pandemic spectrum. J Laryngol Otol 135(5):442–7. 10.1017/s0022215121000992. 10.1017/S0022215121000992 PMC8060545 33827722

